# Stability Study of Multi-Level Grayscales Based on Driving Waveforms for Electrowetting Displays

**DOI:** 10.3390/mi14061123

**Published:** 2023-05-26

**Authors:** Wanzhen Xu, Zichuan Yi, Zhengxing Long, Hu Zhang, Jiaquan Jiang, Liming Liu, Feng Chi, Ding Tan, Huan Wang

**Affiliations:** 1 College of Electronic Information, University of Electronic Science and Technology of China, Zhongshan Institute, Zhongshan 528402, China; 2021024102@m.scnu.edu.cn (W.X.); mikaellzx@163.com (Z.L.); 202021020727@std.uestc.edu.cn (H.Z.); m15820735404@163.com (J.J.); liulmxps@126.com (L.L.); chifeng@semi.ac.cn (F.C.); 2 South China Academy of Advanced Optoelectronics, South China Normal University, Guangzhou 510006, China; 3 School of Information and Optoelectronic Science and Engineering, South China Normal University, Guangzhou 510006, China; 4 Power China Hubei Engineering Co., Ltd., Wuhan 430048, China; td-hb@powerchina.cn; 5 Hydro Electric Power System Engineering Company, Wuhan 430000, China; wanghuan3-js@powerchina-hb.com

**Keywords:** electrowetting, multi-level grayscales, driving waveform, oil backflow

## Abstract

Electrowetting Display (EWD) is a new reflective display with an outstanding performance of color video playback. However, some problems still exist and affect its performance. For instance, oil backflow, oil splitting, and charge trapping phenomena may occur during the driving process of EWDs, which would decrease its stability of multi-level grayscales. Therefore, an efficient driving waveform was proposed to solve these disadvantages. It consisted of a driving stage and a stabilizing stage. First, an exponential function waveform was used in the driving stage for driving the EWDs quickly. Then, an alternating current (AC) pulse signal waveform was used in the stabilizing stage to release the trapped positive charges of the insulating layer to improve display stability. A set of four level grayscale driving waveforms were designed by using the proposed method, and it was used in comparative experiments. The experiments showed that the proposed driving waveform could mitigate oil backflow and splitting effects. Compared to a traditional driving waveform, the luminance stability was increased by 8.9%, 5.9%, 10.9%, and 11.6% for the four level grayscales after 12 s, respectively.

## 1. Introduction

With the continuous progress of science and technology, display technology has developed rapidly, including EWDs [1,2,3,4,5]. As a new type of display device based on electrowetting (EW) technology, EWDs are widely used in wearable electronic devices, electronic tags, and other fields due to their low power consumption, wide viewing angle, and excellent readability in sunlight [6,7,8]. Therefore, EWDs have a high development value and broad development prospects [9].

EW technology was proposed by G. Beni as early as 1981 [10]. In 2003, Hayes prepared an EWD based on pixels [11,12]. The performance of EWDs introduced in 2004 went even further; the luminance of the EWDs was four times that of conventional reflective liquid crystal displays (LCDs) [13]. In 2011, a method of filling different colored oil into sub-pixels was achieved for realizing a single-layer, multi-color EWD [14]. Despite tremendous progress in the development of EWDs, some defects, such as oil splitting, oil backflow, charge trapping, and hysteresis effect [15,16,17,18,19,20], have a bad effect on the display quality. Regarding display principles, EWDs can be improved from interface materials, pixel structures, fluid motion mechanisms, and driving waveforms [21,22,23,24,25]. Many scholars have contributed to improving the performance of EWDs mainly in terms of display principles and driving waveform design, which can be reflected by grayscale stability, and the grayscale stability is affected by oil splitting and oil backflow [26,27,28]. Oil splitting is mainly caused by a sudden change in the driving voltage [29,30]. Therefore, a linear function waveform and an exponential function waveform were proposed to solve this problem [31,32]. Oil backflow is a phenomenon of decreasing aperture ratio caused by charge trapping, which can trap charges in an insulating layer of the EWDs [33]. In order to resolve oil backflow, a driving waveform with a reset signal was proposed for releasing captured charges [34]. These excellent driving waveforms provided inspiration and precious experience for driving waveform design. However, EWDs still do not obtain good stability at multi-level grayscales.

In this paper, based on the analysis of the driving principle, a combined driving waveform was proposed to improve the stability of multi-level grayscales. An exponential function waveform was used to decrease sudden changes in the driving voltage, which can prevent oil splitting. According to charge trapping theory, an AC pulse signal waveform can be used to release trapped charges, which can improve the stability of EWDs.

## 2. Driving Principle of EWDs

Principle of EWDs

**Figure 1:**
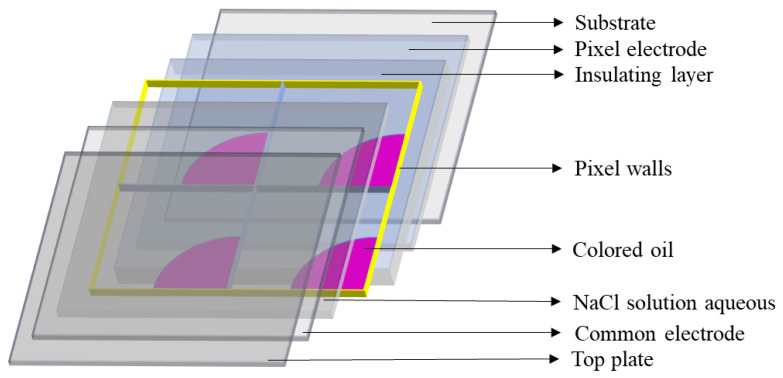
A three-dimensional schematic diagram of pixels in EWDs. It is composed of a substrate, a pixel electrode, an insulating layer, pixel wall, colored oil, NaCl aqueous solution, and a common electrode.

EW technology means that the wettability of droplets on a substrate can be changed by applying a voltage [35,36,37]. Based on the EW principle, the EWD structure can be designed, whose main component is pixels; each pixel consists of a substrate, a pixel electrode, an insulating layer, a pixel wall, colored oil, NaCl solution, a common electrode, and a top plate [2,38,39,40,41]. Its structure is shown in Figure 1.

**Figure 2:**
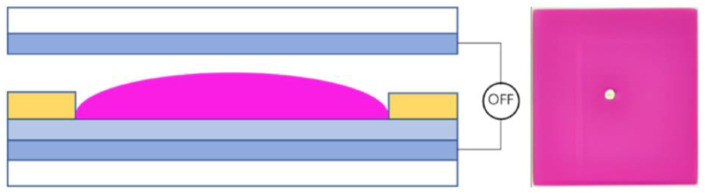
A two-dimensional structure diagram and planform of a pixel in an EWD when no voltage is applied.

**Figure 3:**
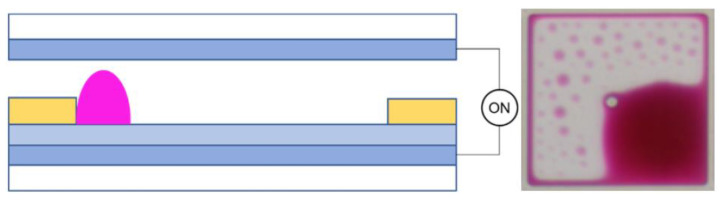
A two-dimensional structure diagram and planform of a pixel in an EWD when a voltage is applied.

In terms of EWD parameters, the threshold voltage, contact angle, and aperture ratio are used to explain the driving process. The threshold voltage of EWDs is the minimum voltage at which the luminance begins to change during the driving process. When a voltage applied between two electrodes is less than the threshold voltage, the insulating layer appears hydrophobic, and colored oil is laid flat in the pixel, as shown in Figure 2. The original equilibrium state of the system is broken, and the solid–liquid contact angle gradually decreases when the voltage between the two electrodes gradually increases. The wettability of the insulating layer is changed, and it appears oleophobic. Thus, the colored oil is pushed to a corner of the pixel by the NaCl aqueous solution, as shown in Figure 3. The contact area between the shrinking oil and the hydrophobic insulating layer is gradually decreased, and eventually, the entire system can reach a new equilibrium state. When the colored oil is pushed away, the ratio of the uncovered area of the colored oil to the area of the pixel is called the aperture ratio, which reflects the luminance of EWDs. The contact angle is another critical parameter in measuring the performance of EWDs, which is a tangency angle at the intersection of solid, liquid, and gas phases. The famous Lippmann–Young Equation derived in 1875 can be used to describe the relationship between the contact angle and applied voltage [42], as shown in Equation (1).

(1) $$\cos\theta_{V}-\cos\theta_{0}=KV^{2}$$

$\theta_{0}$ and $\theta_{V}$ are the contact angles before and after the application of a driving voltage, respectively, and K is a constant related to the material and structure of the EWDs. Ideally, the square of the driving voltage is proportional to the contact angle. However, due to factors such as oil backflow and charge trapping, the Equation (1) can be optimized as Equation (2); $V_{G}$ is the voltage generated by the charge trapping.

(2) $$\cos\theta_{V}-\cos\theta_{0}=K{\left(V-V_{G}\right)}^{2}$$

## 3. Experimental Results and Discussion

### 3.1. Experimental Platform

**Figure 4:**
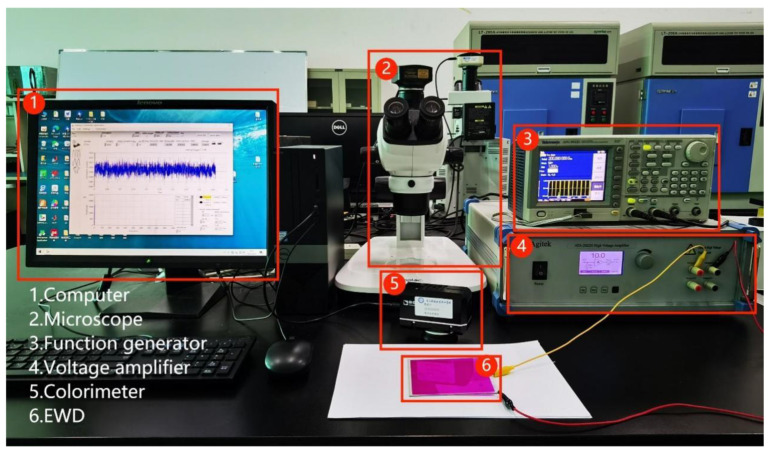
The experimental platform for evaluating the performance of EWDs. It was composed of a computer, a microscope, a function generator, a voltage amplifier, a colorimeter, and an EWD. The EWD was the testing object. The computer was used to design the waveforms. The microscope was used to observe the microscopic forms of the EWD. The function generator and voltage amplifier were used to generate the driving waveforms. The colorimeter was used to measure the luminance of the EWD.

In this experiment, an integrated experiment platform was built to measure and record luminance and response time values, which consisted of a computer, a function generator, a voltage amplifier, a colorimeter, and a microscope. The entire system could be operated as follows. First, driving waveforms were designed on the computer by using professional software (Matlab R2019a). A binary file would be generated, which would describe driving waveforms, and then the file would be stored in the function generator. Due to the limited output voltage of the function generator, the voltage amplifier was used to amplify the driving waveform for driving the EWDs. During the driving process, the colorimeter was applied to measure and record the luminance of the EWDs, and the microscope was used to observe the colored oil in the pixels. The parameters of the instruments used above are shown in Table 1, and the whole experiment platform is shown in Figure 4.

**Figure 5:**
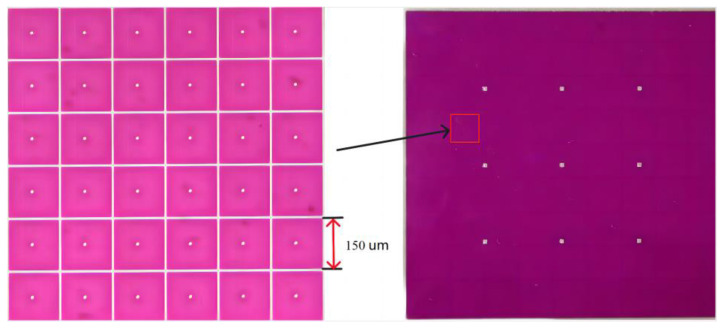
The EWD used for evaluating the display performance.

In the experiment, the EWD used for testing was designed and manufactured by us, as shown in Figure 5. The parameters of the EWD are shown in Table 2.

### 3.2. Proposed Driving Waveforms

**Figure 6:**
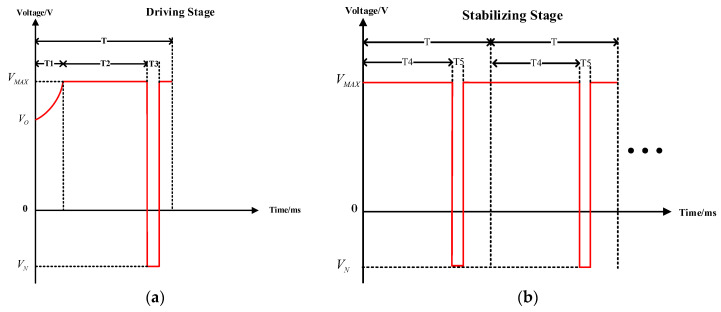
Schematic diagrams of the proposed driving waveform. (**a**) The diagram of the driving stage. $V_{MAX}$ was a voltage at which the pixel could be driven to the target grayscale, and it was also called a target driving voltage. $V_{N}$ was a negative voltage, which could release trapped charges. $V_{0}$ was an initial rising voltage. T was the duration of a display cycle. $T_{1}$ was the duration of the exponential function waveform. $T_{2}$ was the duration of the DC driving process. $T_{3}$ was the duration of the negative voltage in the driving stage. (**b**) The diagram of the stabilizing stage. $T_{4}$ was the duration of the DC driving process in the stabilizing stage. $T_{5}$ was the duration of the negative voltage in the stabilizing stage.

In order to drive the EWDs to realize maximum luminance and stable performance at a specific grayscale, a driving waveform based on an exponential function and an AC square wave was proposed, including a driving stage and a stabilizing stage, as shown in Figure 6.

In the driving stage, the proposed waveform was mainly based on an exponential function and an AC square. The exponential function was used to drive the EWDs to achieve maximum luminance quickly. The AC square was used to release trapped charges, which can reduce the effect of $V_{G}$ in Equation (2) and suppress oil backflow in the driving stage. The equation of the exponential function is shown in Equation (3).

(3) $$U=\left(V_{0}-1\right)+e^{at}$$

U is the driving voltage in real-time. a is a time constant. $V_{0}$ is the initial voltage. It could be observed that a affected the rising rate of the driving voltage. Therefore, $T_{1}$ could keep a constant by adjusting a when the target driving voltage was different. In the stabilizing stage, the proposed driving waveform was composed of many display cycles. Each display cycle had an AC square, which was used to keep the EWD’s display stable.

### 3.3. Parameter Optimization of the Driving Stage

**Figure 7:**
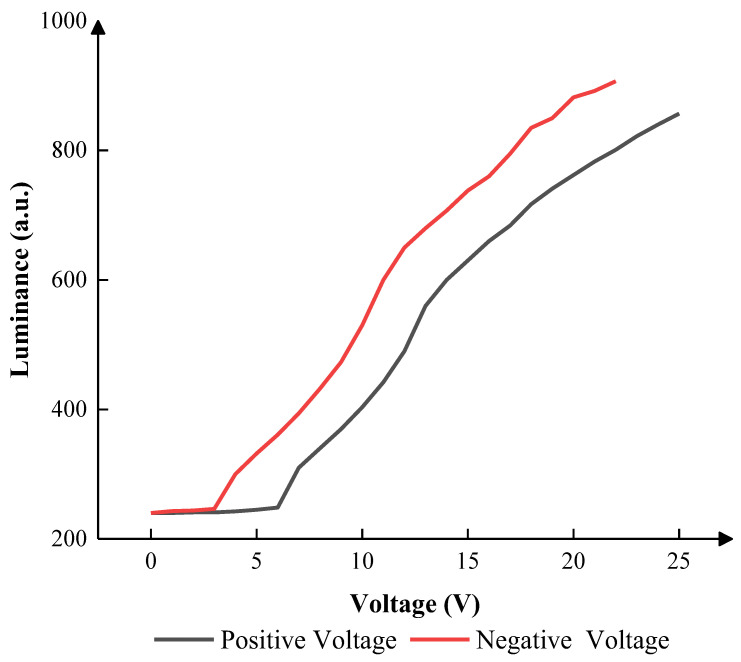
The luminance of the EWD, which was applied to different DC voltages. The luminance was 240 when the positive DC voltage was less than 7 V. The luminance was 243 when the negative DC voltage was less than 4 V. The maximum luminance was obtained when the DC voltage was 25 V.

In the driving stage, parameters would be optimized according to the relationship between the luminance and the driving voltage. Therefore, to obtain the relationship, a DC waveform was designed, which was set to 0 V to 25 V, and the maximum voltage was set to 25 V to protect the EWDs. Figure 7 shows the corresponding relationship between the DC driving voltage and the luminance. The luminance showed an upward trend, and the growth rate decreased gradually. The EWD could not be driven when the positive voltage was lower than 7 V. The EWD also could not be driven when the negative voltage was lower than 4 V. Therefore, the positive and negative threshold voltage amplitude were set to 7 and 4 V, respectively.

**Figure 8:**
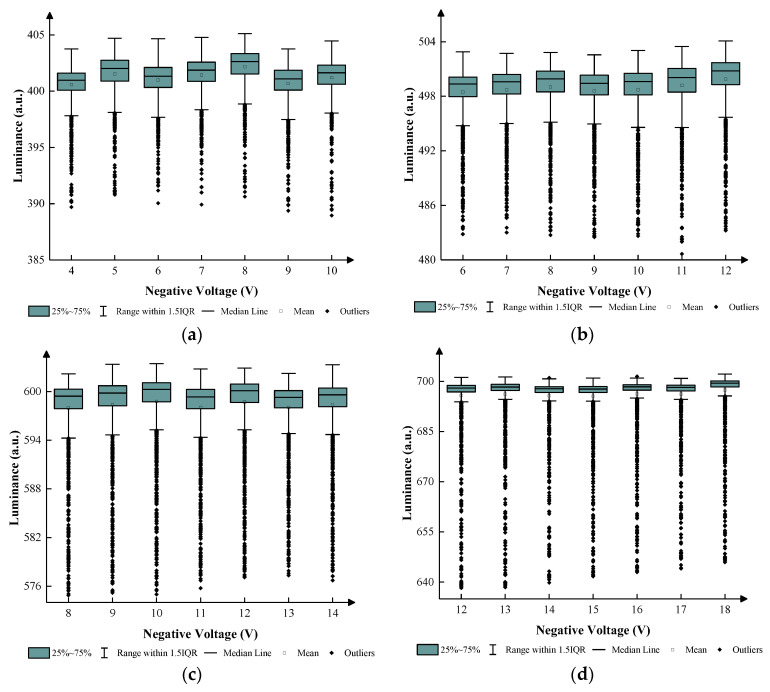
The luminance of the EWD when different negative voltages were applied. The median line, mean, and other parameters reflect the distribution of the luminance. (**a**) Relationship between luminance values and negative voltages at the first grayscale. (**b**) Relationship between luminance values and negative voltages at the second grayscale. (**c**) Relationship between luminance values and negative voltages at the third grayscale. (**d**) Relationship between luminance values and negative voltages at the fourth grayscale.

According to Figure 7, the maximum luminance was 857 when 25 V-positive DC voltage was applied. In this paper, the number of grayscales was set to four as an illustrative example, and the luminance of four grayscales was set to 400, 500, 600, and 700. The target driving voltages were set to 10 V, 12 V, 14 V, and 18 V, and the luminance values were 403, 490, 600, and 717, respectively. The display cycle T was set to 20 ms because 50 Hz was a usual frequency that could not be recognized by eyes. $V_{N}$ was used to release trapped charges. $T_{1}$ was set to 5 ms. $T_{2}$ was set to 13 ms to drive the EWD at the maximum luminance. $T_{3}$ was set to 0.25 ms to release trapped charges, which could prevent oil backflow caused by charge trapping. According to Equation (3), $V_{0}$ was set to 7 V, 9 V, 11 V, and 14 V to obtain driving waveforms of the same shape in four grayscales when $T_{1}$ was 5 ms. The range of $V_{N}$ was set to 4–10 V, 6–12 V, 8–14 V, and 12–18 V. The luminance between different grayscales and $V_{N}$ in 200 ms is shown in Figure 8.

**Figure 9:**
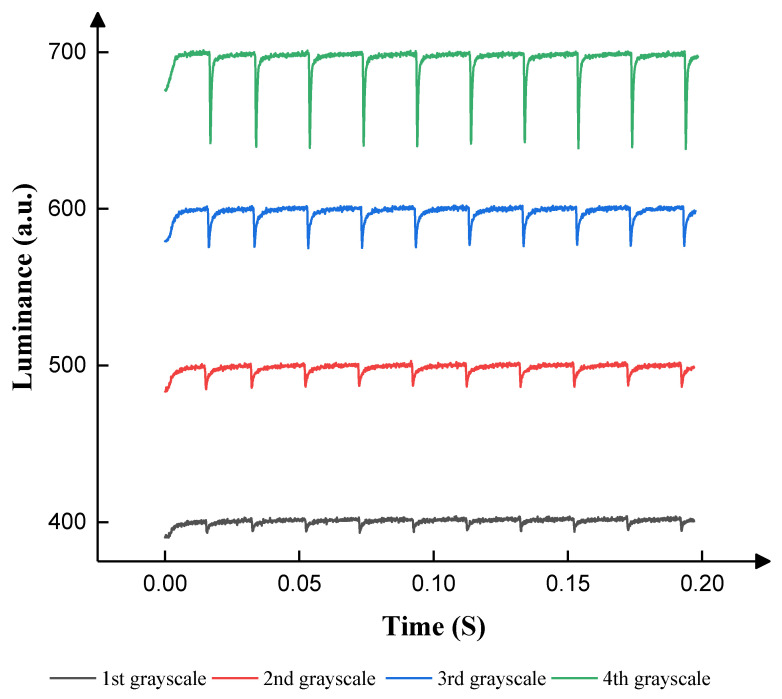
The luminance of the EWD when different amplitude negative voltages were applied.

According to Figure 8, the luminance of the EWD barely changed when $V_{N}$ was lower than $V_{MAX}$. In the first grayscale, the median luminance was maintained at approximately 402 when $V_{N}$ was set to 4–10 V. The average luminance was slightly less than the median luminance but remained above 400. The interquartile range (IQR) was maintained at approximately 3. The difference between the burr signal and the stable luminance was within 12. In the second grayscale, the median luminance was maintained at approximately 500 when $V_{N}$ was set to 6–12 V. The average luminance was slightly less than the median luminance, but it was very close to 500. The IQR was maintained at approximately 3. The difference between the burr signal and the stable luminance was within 18. In the third grayscale, the median luminance was maintained at approximately 599 when $V_{N}$ was set to 8–14 V. The average luminance was slightly less than the median luminance, but it was very close to 598. The IQR was maintained at approximately 4. The difference between the burr signal and the stable luminance was within 25. In the fourth grayscale, the median luminance was maintained at approximately 698 when $V_{N}$ was set to 12–18 V. The average luminance was slightly less than the median luminance, but it was very close to 694. The IQR was maintained at approximately 3. The difference between the burr signal and the stable luminance was within 62. The applied negative voltage hardly influenced the luminance. Therefore, $V_{N}s$ were set to 4 V, 6 V, 8 V, and 12 V in different grayscales. Figure 9 demonstrates the relationship between the driving time and the luminance of the EWD when negative voltages were set to 4 V, 6 V, 8 V, and 12 V in four grayscales, respectively.

### 3.4. Testing of the Stabilizing Stage

**Figure 10:**
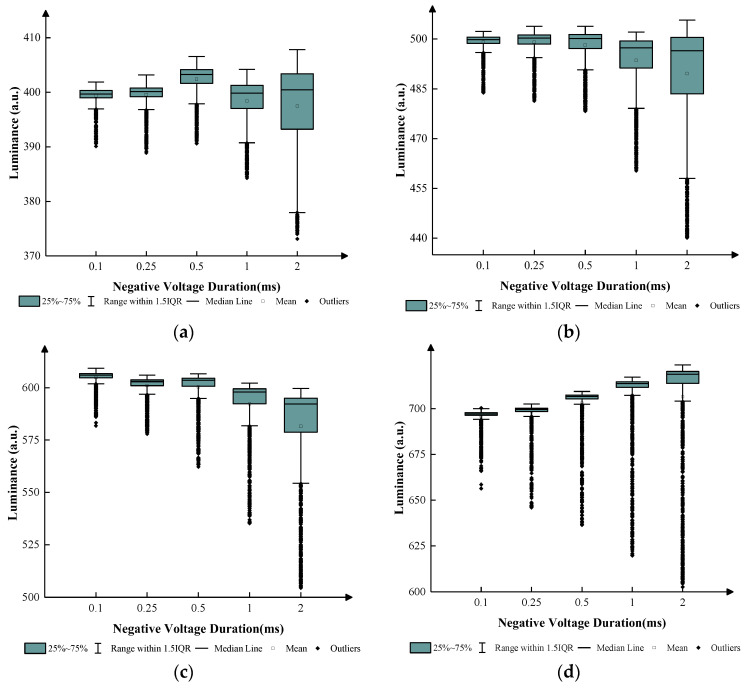
The luminance of the EWD within 200 ms when negative voltages were applied. The median line, mean, and other parameters reflect the distribution of the luminance. (**a**) Relationship between luminance values and negative voltages at the first grayscale. (**b**) Relationship between luminance values and negative voltages at the second grayscale. (**c**) Relationship between luminance values and negative voltages at the third grayscale. (**d**) Relationship between luminance values and negative voltages at the fourth grayscale.

In the driving stage, the applied negative voltage was evaluated to obtain the optimum negative voltage. The luminance would decrease when a negative voltage was applied. $T_{3}$ was set to 0.25 ms due to the limitation of the display cycle. In the stabilizing stage, only $T_{4}$ and $T_{5}$ would be considered. $T_{4}$ was set to 15 ms to keep a stable luminance. $T_{5}$ could be set to 0.1 ms, 0.25 ms, 0.5 ms, 1 ms, and 2 ms in four grayscales, respectively. Negative voltages were set to 4 V, 6 V, 8 V, and 12 V, respectively. Figure 10 shows the relationship between luminance and $T_{5}$ in different grayscales within 200 ms.

According to Figure 10, the luminance distribution of the EWD changed when $T_{5}$ was changed. As $T_{5}$ increased, the luminance fluctuation also increased. In the first grayscale, the median luminance of the EWD maintained at approximately 401 when $T_{5}$ was set to 0.1 ms, 0.25 ms, 1 ms, and 2 ms. The average luminance declined below 400 when $T_{5}$ was set to 1 and 2 ms. The IQR increased from 2 to 10 when $T_{5}$ increased; therefore, the display stability became worse. The EWD had better stability within 200 ms when $T_{5}$ was lower than 2 ms. In the second grayscale, the median luminance and average luminance maintained at approximately 500 when $T_{5}$ was set to 0.1 ms, 0.25 ms, and 0.5 ms. The median luminance and average luminance decreased below 500 when $T_{5}$ was set to 1 ms and 2 ms. The IQR increased from 2 to 20 when $T_{5}$ increased. The EWD had better stability within 200 ms when $T_{5}$ was lower than 2 ms. In the third grayscale, the median luminance and average luminance maintained at approximately 600 when $T_{5}$ was set to 0.1 ms, 0.25 ms, and 0.5 ms. The median luminance and average luminance decreased below 600. The IQR increased from 2 to 20 when $T_{5}$ increased. The EWD had better stability within 200 ms when $T_{5}$ was smaller than 1 ms. In the fourth grayscale, the median luminance and average luminance maintained at approximately 700 when $T_{5}$ was set to 0.1 and 0.25 ms. The median luminance and average luminance increased over 600 when $T_{5}$ was set to 0.5 ms, 1 ms, and 2 ms. The IQR increased from 2 to 10 when $T_{5}$ increased. Therefore, the EWD had better stability within 200 ms when $T_{5}$ was set to 0.1 ms, 0.25 ms, and 0.5 ms in four grayscales.

**Figure 11:**
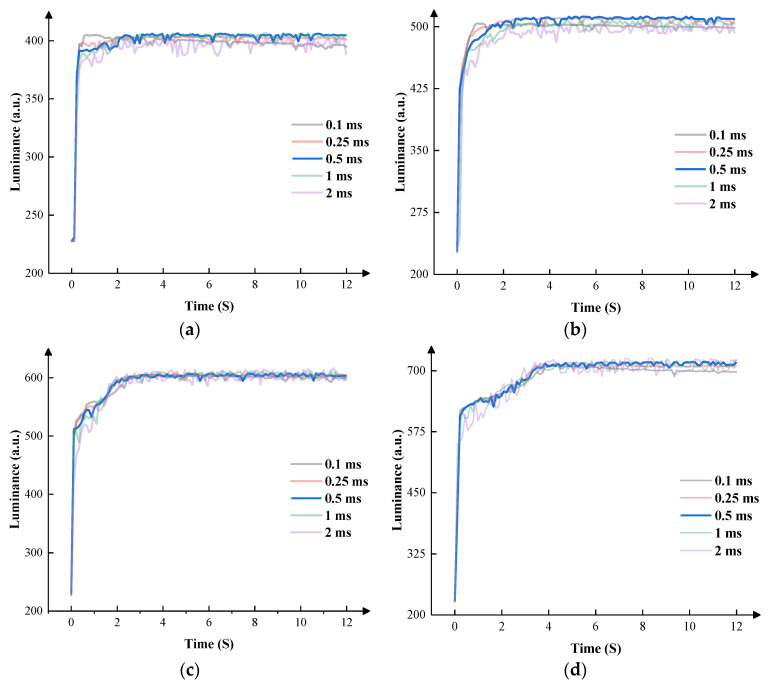
The luminance of the EWDs during a long duration when different negative voltages were applied. (**a**) Relationship between luminance values and the negative voltage at the first grayscale. (**b**) Relationship between luminance values and the negative voltage at the second grayscale. (**c**) Relationship between luminance values and the negative voltage at the third grayscale. (**d**) Relationship between luminance values and the negative voltage at the fourth grayscale.

The stability of the EWDs could not be completely proven in a short duration. The relationship between driving time and voltage in a long display duration must be considered in terms of practicality. Due to the charge trapping effect, the insulating layer of the EWD stored charges continuously when a unipolar voltage was applied for a long duration, which caused oil backflow and affected the display performance. Therefore, the negative voltage was designed to suppress the oil backflow. Figure 11 demonstrates the relationship between the driving time and the luminance of the EWDs for a long duration when the negative voltage was set to 4 V, 6 V, 8 V, and 12 V in four grayscales, respectively.

**Figure 12:**
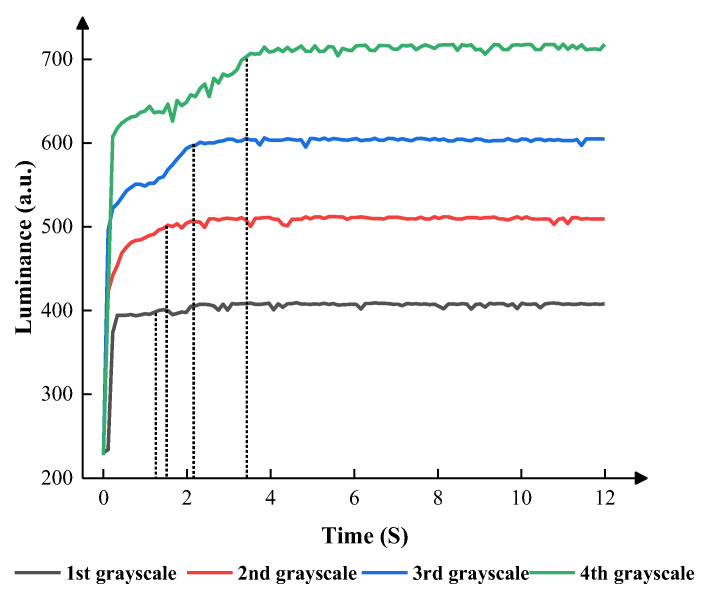
The luminance of the EWDs within a long duration when the best parameters were applied. In the first grayscale, $V_{N}$ was set to 4 V, and $T_{5}$ was set to 0.5 ms. In the second grayscale, $V_{N}$ was set to 6 V, and $T_{5}$ was set to 0.5 ms. In the third grayscale, $V_{N}$ was set to 8 V, and $T_{5}$ was set to 0.5 ms. In the fourth grayscale, $V_{N}$ was set to 12 V, and $T_{5}$ was set to 0.5 ms.

According to Figure 11, $T_{5}$ was an essential part of the EWD’s stability. In the first grayscale, the EWDs obtained the shortest response time when $T_{5}$ was set to 0.1 ms. Oil backflow occurred when $T_{5}$ was set to 0.1 ms and 0.25 ms, which affected the display stability. Therefore, the EWD could display steadily when $T_{5}$ was set to 0.5 ms and 1 ms. Compared to 1 ms, the EWD could obtain a higher luminance and a higher response speed when $T_{5}$ was set to 0.5 ms. In the second grayscale, the EWD obtained the shortest response time when $T_{5}$ was set to 0.1 ms. Oil backflow occurred when $T_{5}$ was set to 0.1 ms. Therefore, the EWDs displayed steadily when $T_{5}$ was set to 0.25 ms and 0.5 ms. Compared to 0.25 ms, the EWDs could obtain a higher luminance when $T_{5}$ was set to 0.5 ms. In the third grayscale, the EWDs obtained the shortest response time when $T_{5}$ was set to 2 ms and obtained the longest response time when $T_{5}$ was set to 0.1 ms. Oil backflow occurred when $T_{5}$ was set to 0.1 ms. Therefore, the EWDs displayed steadily when $T_{5}$ was set to 0.5 and 1 ms. Compared to 0.25 ms, the EWDs could obtain a higher luminance and better stability when $T_{5}$ was set to 0.5 ms. In the fourth grayscale, the EWD obtained the shortest response time when $T_{5}$ was set to 2 ms. Oil backflow occurred when $T_{5}$ was set to 0.1 ms and 0.25 ms. Therefore, the EWDs displayed steadily when $T_{5}$ was set to 0.5 and 1 ms. Compared to 1 ms, the EWDs could obtain a higher luminance and a shorter response time when $T_{5}$ was set to 0.5 ms. According to Figure 12, the response time of the EWD increased gradually as the grayscale increased.

### 3.5. Performance of the Proposed Waveform

**Figure 13:**
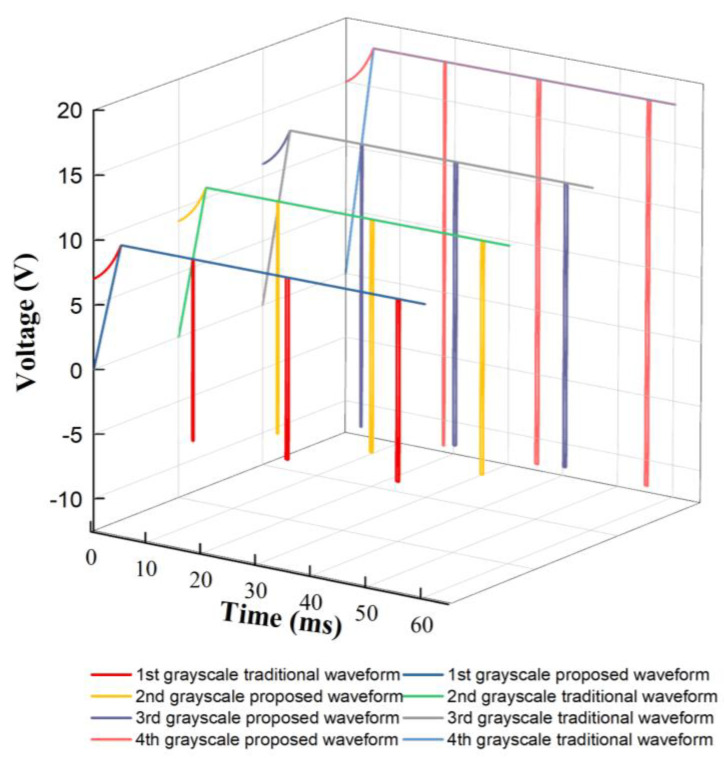
Proposed driving waveforms and traditional driving waveforms of different grayscales for performance comparison.

As shown in Figure 13, traditional driving waveforms were used to compare performance with the proposed driving waveform. The rising duration of the driving stage was set to 5 ms, and the target driving voltage $V_{MAX}$ was set to 10 V, 12 V, 14 V, and 18 V. The initial voltage was set to 0 V.

As shown in Figure 13, traditional driving waveforms were used to compare display performance with the proposed driving waveform. The rising duration of the driving stage was also set to 5 ms, and the target driving voltages were set to 10 V, 12 V, 14 V, and 18 V in four grayscales. The initial voltage was set to 0 V.

**Figure 14:**
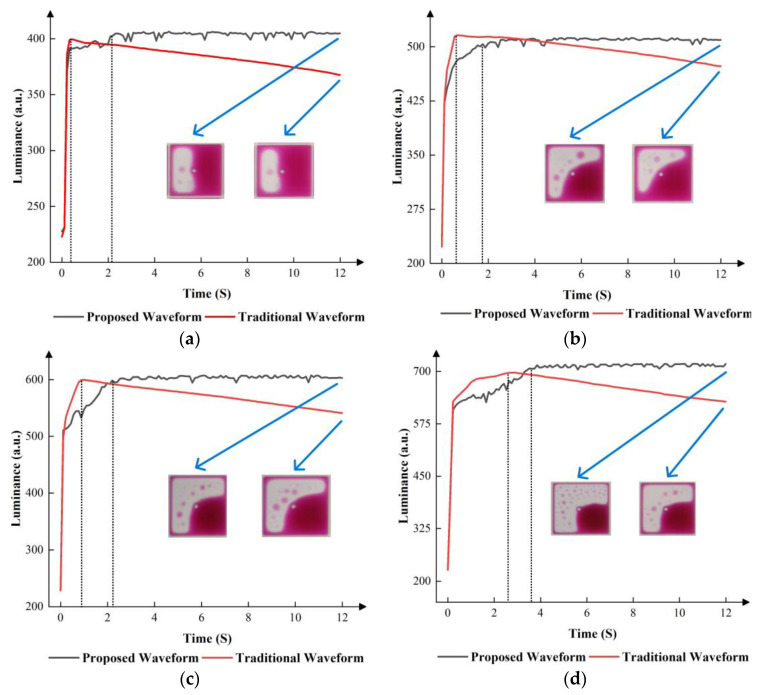
Luminance curves of different driving waveforms in different grayscales. (**a**) Luminance curves of different driving waveforms in the first grayscale. (**b**) Luminance curves of different driving waveforms in the second grayscale. (**c**) Luminance curves of different driving waveforms in the third grayscale. (**d**) Luminance curves of different driving waveforms in the fourth grayscale.

According to Figure 14, compared to the traditional driving waveform, the proposed driving waveform had a better performance. In the first grayscale, the luminance of the proposed driving waveform steadily increased to approximately 400 and maintained at 400. Although the traditional driving waveform had a shorter response time, the luminance started to decline after reaching 400. In the second grayscale, the luminance of the proposed driving waveform steadily increased to approximately 500 and was maintained at 510. The luminance of the traditional driving waveform started to decline after reaching 520. In the third grayscale, the luminance of the proposed driving waveform steadily increased to approximately 600 and maintained at 600. The luminance of the traditional driving waveform started to decline after reaching 600. In the fourth grayscale, the luminance of the proposed driving waveform steadily increased to approximately 700 and maintained at 700. The luminance of the traditional driving waveform started to decline after reaching 700. Generally, the proposed driving waveform solved the oil backflow, which was caused by charge trapping. Compared to the traditional driving waveform, the EWD had better stability by applying the proposed driving waveform.

## 4. Conclusions

In order to achieve a stable display performance of multi-level grayscales, a new combined driving waveform was proposed in this paper, which was based on an exponential function and AC voltage. Compared to the traditional driving waveform, the effectiveness of the proposed driving waveform was proven. The proposed driving waveform could suppress oil backflow at different grayscales and reduce the luminance oscillation of the EWDs. In addition, the proposed driving waveform could be adapted to drive the EWDs with more than four grayscales. In summary, the proposed driving waveform provided a reference value for improving multi-level display performance of the EWDs.

## Figures and Tables

**Table 1:** Parameters of the experimental instruments.

Name	Model	Manufacturers	Region	Region
Computer	H430	Lenovo	Beijing	China
Function generator	AFG3022C	Tektronix	Beaverton	USA
Voltage amplifier	ATA-2022H	Agitek	Xian	China
Microscope Colorimeter	SZ680 Arges-45	Cnoptec Admesy	Chongqing Ittervoort	China Netherlands

**Table 2:** Parameters of the EWD panel.

Panel Size	Oil Color	Resolution	Pixel Size	Pixel Wall Hight	Insulating Layer Thickness	Electrode Plates Thickness
10 × 10 cm	magenta	320 × 240	150 × 150 μm	18 μm	1 nm	2.5 nm

## Data Availability

Data is contained within the article.
